# Women rights from Islamic perspectives: navigating rights, challenges and contemporary perspectives

**DOI:** 10.3389/fsoc.2025.1727894

**Published:** 2026-01-14

**Authors:** Duman Saifullin, Samet Okan, Askar Akimkhanov

**Affiliations:** Egyptian University of Islamic Culture Nur-Mubarak, Almaty, Kazakhstan

**Keywords:** challenge, Islam, law, patriarchy, women’s rights

## Abstract

This paper examines the role and rights of women through Islamic lens, tackling modern issues that obstruct gender parity. It highlights the historical progress and governance of women in the Islamic context, drawing attention to the differences in interpretations and practices across various cultures and nations. The research seeks to demonstrate that Islam does not oppress women but instead grants them rights bestowed by Allah, challenging baseless claims against the faith. The study identifies and scrutinizes the barriers women encounter in obtaining equal educational opportunities, influenced by a complex interaction of social norms, conservative religious views, and systemic hurdles. It also explores the effects of religious texts and traditions on gender equality, acknowledging the role of feminist movements in advancing women’s rights within Muslim communities. Furthermore, the article emphasizes the ongoing conversations about women’s rights in Islam, illustrating the dynamism of Islamic jurisprudence and its ability to adapt to changing social circumstances. It stresses the necessity for critical evaluations of conventional interpretations, advocating for a reassessment that prioritizes gender equity and justice within Islamic teachings. Ultimately, the research reveals the complex and layered nature of women’s rights in the Islamic world, highlighting the significance of harmonizing universal human rights principles with cultural norms to empower women and uphold their inherent rights.

## Introduction

The rights of women within the framework of Islam have emerged as a prominent subject of modern discourse, encompassing matters of equality, justice, and, indeed, textual interpretation. The critics assert that conventional Islamic customs and laws, including limitations on divorce, exhibit discriminatory tendencies towards women. They highlight that in certain Muslim communities, women encounter biases in educational access, attendance, and participatory opportunities. Conversely, Islamic apologists contend that Islam bestows adequate rights and protections in contemporary society. They argue that Islam assures women the entitlement to education, imposes restrictions on property rights, and endorses their engagement in commercial enterprises. Furthermore, they underscore that Islam forbids women from independently earning income and mandates that such income be treated with dignity (Maloko and Chotban, 2024). To improve the situation modern Muslim activists advocate for a reexamination of Islamic texts to foster enhanced justice and equality for women. They assert that historical and cultural influences frequently affect the interpretation of Islamic narrations, and that it is feasible to develop more egalitarian methodologies that align with the fundamental principles of the Islamic faith. The pivotal aspect of this discourse is the interpretation of the Quran and Sunnah. Traditional interpretations often resonate with patriarchal norms, while progressive scholars propose alternative interpretations that elaborate on the context and ethos of justice inherent in Islam. They contend that numerous practices traditionally viewed as Islamic are the result of cultural customs rather than rigorously defined regulations (Khofiyya et al., 2024).

A 2013 global study by the Pew Research Center, based on over 38,000 in-person interviews across 39 such countries revealed issues of women’s rights in the Muslim world (Lo and Seltzer, 2015). Gutmann and Voigt (2015) contribute to the scholarly discourse on gender equality within Islamic contexts by developing an Islamic State Index. They evaluate women’s rights through both legal (de jure) and practical (de facto) frameworks. Legal metrics encompass economic, employment, inheritance, and property rights, whereas practical rights are assessed utilizing the CIRI Human Rights Dataset. Their research indicates that a heightened adherence to Islamic principles is associated with increased discrimination against women, particularly in relation to social rights. The study encompasses both nations with Muslim majorities and those wherein other faiths, such as Christianity, predominate (Gutmann and Voigt, 2015).

The discourse surrounding women’s rights within the framework of Islamic law has garnered significant attention in contemporary society, reflecting a complex interplay between tradition, interpretation, and modernity. As societies evolve and the role of women continues to expand across various spheres, the principles of Islamic jurisprudence emerge as a critical lens through which to examine gender equity and rights. The interpretations of Islamic texts have, at times, contributed to disparities in legal and social status between men and women. The challenge lies in discerning between cultural norms that have been historically associated with Islam and the divinely ordained principles themselves (Meidina, 2022). Contemporary Islamic scholars and activists are increasingly engaging in critical analyzes of traditional interpretations, advocating for a re-evaluation that prioritizes gender equity. This involves revisiting classical texts with an eye contextual understanding and emphasizing spirit of justice and compassion inherent in Islamic teachings. The ongoing dialogue concerning women’s rights in Islam highlights the dynamism of Islamic jurisprudence and its capacity to adapt to evolving social contexts. By critically examining traditional interpretations and prioritizing the principles of justice and equality, Muslim societies can strive to create legal and social frameworks that empower women and uphold their fundamental rights within the framework of Islamic law (Panjwani, 2020).

The subject of women’s rights within the context of Islam necessitates comprehensive scholarly inquiry intended to reinstate justice and counter unfounded allegations. The objective is to elucidate that Islam does not serve as a system that subjugates women; on the contrary, it bestows upon them the rights conferred by Allah. The primary objective of this research is to elucidate the role and rights of women in an Islamic perspective. The article identifies and analyzes the challenges and obstacles that women continue to face in accessing equal educational opportunities. These obstacles are shaped by a complex interplay of social norms, conservative religious interpretations, and socio-cultural and structural challenges. To achieve complete gender equality, it is imperative to address these impediments and dismantle the structural barriers that impede women’s advancement. The paper addresses the evolving roles and rights of women in the modern era, particularly from an Islamic perspective. It highlights that, despite advancements, women often face social injustices and are sometimes viewed as inferior beings in various societies.

## Literature review

The multitude of scholarly publications studies women’s rights within Islam, primarily scrutinizing the legal status of women under Sharia law, their entitlements to education, employment, political engagement, and familial dynamics. Ahmed Salman, Alotaibi Hessa and Arfah emphasize historical developments during the 20th century and governance of women within the Islamic realm, encompassing the variances in interpretations and practices across different nations and cultures. The paper of Begum recognizes the impact of religious texts and traditions on gender equality, along with the contribution of feminist movements in promoting women’s rights within Muslim communities. Significantly, scholarly inquiry often centers on the degree to which women’s rights are upheld and safeguarded in the Islamic world, illustrating the intricate and multifaceted essence of the subject matter. The researchers such as Khofiyya, Maloko and others investigate the discrepancies between universal human rights principles and cultural norms, as well as identifying methods to reconcile these two systems. Notwithstanding the conservative corpus of traditional religious scholarship, the discourse surrounding women’s rights in Islam still persists as a controversial topic.

## Research methods

This study employs diverse methods to explore women’s rights through an Islamic lens and to identify present-day obstacles. The researchers conduct a thorough survey of scholarly works examining women’s rights within Islamic contexts. This includes a comprehensive analysis of women’s legal status under Sharia, their access to education, work, and political involvement, alongside family structures. The study recognizes the impact of religious texts and customs on attaining gender equality. It critically analyzes historical interpretations of these texts and their impact on contemporary views of women’s rights in Muslim societies. The authors delve into the part played by feminist movements in championing women’s rights within these communities, highlighting the importance of activism in challenging traditional views and fostering gender equality. The study takes into account the complex interplay between societal norms, conservative religious interpretations, and systemic issues that define the obstacles women face. This analysis is essential for understanding the broader context of women’s rights in Islamic societies. The researchers investigate the disparities between global human rights norms and cultural practices, seeking pathways to reconcile these frameworks. This comparative approach is critical for grasping the challenges and possible strategies for advancing women’s rights in the Islamic world. Together, these methods encourage a deep understanding of women’s rights in Islam, stressing the need for ongoing discussion and critical review of traditional interpretations to promote gender equality and justice.

## Discussion and results

Historically Islam has played a crucial role in elevating the status of women, who historically faced numerous injustices. The Islamic teachings have restored women’s rights, allowing them to reclaim their dignity and rightful place in society. Moreover, Islamic teachings support women’s involvement in both family and community life, thus promoting a balanced view of gender roles in society (Ahmed, 2022). Islam arrival marked a significant change in the perception and treatment of women, aiming to eradicate injustices and promote equality. Moreover, Islam emerged as a liberating force, elevating the status of women and granting them rights such as the ability to marry, divorce, inherit property, and make independent decisions (Arfah, 2024). The Islamic tradition, as derived from the Quran and hadith, asserts that there should be no distinction between the rights of men and women. The women in Islamic medieval ages enjoyed equal rights and status in society, contrasting sharply with the current conditions where many Muslim women face discrimination and limited rights (Mun’im et al., 2024). The women hold a noble and significant position in Islamic law. This is highlighted by the considerable attention given to women’s rights, which are deemed crucial in society. There should be no distinction among people except for their piety.

The active involvement of women in the public sphere during the formative centuries of Islam is substantiated by a plethora of historical documents. For instance, in the era of the Prophet Muhammad (peace be upon him), Khadija bint Khuwaylid emerged as one of the most prominent businesswomen in Mecca and is regarded as a quintessential example of a woman who attained economic autonomy during her era. In the period of Medina, Aisha bint Abu Bakr assumed a significant role in the transmission of hadith, jurisprudential matters, and in the shaping of public sentiment; her contributions to the evolution of Islamic sciences, as numerous companions relied upon her legal opinions, attest to the fact that women operated autonomously within the intellectual domain. Furthermore, the designation of a female scholar, ash-Shifa bint Abdullah, as a market supervisor (muhtasiba) during the caliphate of Umar ibn al-Khattab, concerning the regulation of the marketplace (as-suk), is regarded as an early instance of women’s involvement in governmental affairs (Hafizah, 2024).

In contemporary Muslim nations, the legal and societal standing of women is undergoing progressive development at varying degrees. For instance, in Indonesia, women have been appointed to the Supreme Court (Mahkamah Agung) and fulfill roles as judges within Sharia courts; this advancement is attributable to contemporary legal interpretations that permit women to assume judicial positions within the framework of Islamic schools of law (madhab). In Malaysia, women are actively engaged in the academic councils pertaining to Islamic finance, waqf administration, and fiqh academies. In Morocco, the Moudawana (Family Code) enacted in 2004 has broadened women’s rights concerning marriage, divorce, guardianship, and inheritance, thereby demonstrating that legal reforms aimed at achieving gender equality can be effectively realized through the reinterpretation of Islamic law (Khan, 2021).

Islamic scholars such as Ibn Baz and Ibn Uthaymeen, representatives of the most conservative Islam in modern world, tend to interpret women’s rights by relying on the explicit textual evidence (nass) found within the Quran and Sunnah. They conceptualize gender distinctions as “variations in legal responsibilities” and prioritize the principle of “justice” over that of “equality” as the fundamental tenet. For instance, Ibn Baz’s fatwas advocate for women’s engagement in public spheres, yet necessitate stringent compliance with Sharia regulations (Belhachmi, 2005). These views are challenged by contemporary Muslim intellectuals Amina Wadud, Asma Barlas, and Fatima Mernissi who argue that patriarchal interpretations of Islam stem from culture and history rather than the Qur’an itself. They emphasize that justice, equality, and ethical responsibility are central Qur’anic principles that support women’s full spiritual and social agency. Leila Ahmed and Riffat Hassan highlight how historical contexts shaped gender norms and call for renewed interpretations that reflect the Qur’an’s egalitarian messages. Meanwhile, Khaled Abou El Fadl and Ziba Mir-Hosseini critique authoritarian legal traditions and advocate for reform grounded in the contemporary human rights (Khanom et al., 2024).

Islamic law encompasses women’s rights across multiple domains, namely women have rights related to their faith and worship practices. This includes rights in political participation, education, and employment. Also, they are entitled to rights concerning economic matters, inheritance, marriage, and judicial processes (Maloko and Chotban, 2024). Nevertheless, over the passage of time and influenced by array of cultural and political factors, the role of women in Muslim nations has undergone significant transformation. Consequently, according to Alotaibi Hessa, the present circumstances do not accurately represent the foundational doctrines of Islam, but are, instead, the outcome of historical evolution and socio-cultural changes (Hessa, 2021).

The men and women have complementary roles. Islam rejects the notion of gender superiority, advocating for a balanced view where both genders share responsibilities and rights within the family structure. Qur’an advocates for mutual respect and cooperation between men and women, rather than subjugation (Rizal et al., 2024). Besides Islam advocates for women’s education rights but persistence of traditional and patriarchal values has created barriers to women’s education in Muslim communities. These cultural norms have historically placed heavy burdens on women, limiting their access to educational resources and opportunities. The researchers stress the importance of understanding their rights and the need for societal support to overcome the barriers they face in accessing education (Jawed and Sikka, 2024). In the legal construction of women’s status, women have been prescribed lacking the same abilities and capabilities as men. As such, their status and rights differ, justifying men to be the maintainers of women. By presenting the historical development of women’s status and how women’s legal status is debated in contemporary Muslim societies, Mona Samadi convincingly provides various methods for facilitating change within the Islamic legal theory framework (Samadi, 2021).

The matter of women’s rights within the context of Islam has emerged as a topic of considerable discourse in contemporary society, notwithstanding the assertion that Islam originally championed these rights. To begin with, Islam initially advanced rights such as the right to education, the imposition of limited restrictions, and engagement in economic activities, which represented a progressive advancement for that era. Nevertheless, as time progressed, cultural customs and traditional texts prevalent in various Islamic societies have contributed to the curtailment of these rights. The adherence to patriarchal norms and traditions within diverse Muslim communities has significantly influenced the restriction of women’s rights. Interpretations of texts that mirror societal impacts on gender stereotypes have resulted in discriminatory practices against women in domains such as marriage, divorce, child custody, and instances of violence. Leaders from various scholarly and organizational backgrounds present differing interpretations that engender confusion and permit arguments to be utilized in support of diverse, often contradictory practices (Samadi, 2021).

### Islam is a religion that advocates for women’s rights

Islam is a faith that champions the rights of women. This notion may appear paradoxical to some individuals, particularly in light of prevalent stereotypes and biases. Nevertheless, upon examining the foundational texts of Islam, Quran and Sunnah, it becomes evident that the Islamic tradition bestows upon women a comprehensive array of rights and opportunities (Abdullah and Hamzah, 2024). Women, similar to their male counterparts, are mandated to pursue education and cultivate their intellectual faculties. They possess the right to own property, receive inheritance, and engage in business activities. Islam safeguards the rights of women within the institution of marriage, encompassing the right to seek divorce and receive financial sustenance. Furthermore, Islam imposes the obligation upon men to provide financial support to women, be they mothers, wives, daughters, or sisters. This delineation implies that a woman is not compelled to engage in employment and generate income if she has a husband, father, or brother capable of fulfilling her financial needs. Nonetheless, she retains the unequivocal right to work and manage her earnings at her own discretion (Warren, 2011). The Islamic tradition also accords significant importance to the role of women as mothers and educators of forthcoming generations. The upbringing of children is deemed one of the most crucial and esteemed responsibilities of a woman, and her contributions to societal development are acknowledged as profoundly valuable (Begum et al., 2024). In the Islamic tradition, the pursuit of education is regarded as an inherent right bestowed upon every individual, irrespective of gender.

The Quran and Sunnah underscore the significance of knowledge acquisition, thereby establishing it as an obligation for both males and females. Historically, women within the Islamic realm have rendered substantial contributions to the fields of science, literature, and education, thereby illustrating their active engagement in the intellectual fabric of society. Islamic scholars contend that affording women equitable educational opportunities plays a pivotal role in the overall advancement of society. An educated woman is better equipped to nurture her offspring, contribute effectively to the economy, and partake actively in civic affairs (Jawed and Sikka, 2024). The Quran does not impose any restrictions upon the educational entitlements of individuals, regardless of gender, within the framework of Islam. The Quran significantly underscores the principle that the individuals most revered in the sight of Allah are those who commit themselves to a virtuous and pious manner of living (Firdaus and Arifin, 2018).

All these facts elucidate Islam as a religion that underscores the rights of women. Islam bestows particular significance upon the role of motherhood, venerating mothers and accentuating their crucial function in nurturing forthcoming generations. Within the Islamic tradition, reverence and care for mothers are regarded as among the highest of virtues. This fortifies the status of women within society and acknowledges their invaluable contributions to familial and societal development. Islam categorically prohibits any form of violence and abuse directed towards women. The safeguarding of women from both physical and psychological violence constitutes a foundational principle of Islamic doctrine. Adherents of Islam are duty-bound to treat women with respect, kindness, and compassion. Islam grants women a plethora of rights and privileges aimed at preserving their dignity, ensuring their well-being, and broadening their opportunities across various realms of life (Bishin and Cherif, 2017). Islam assigns great importance to the role of women, granting them a distinguished status within society. The compilation of the arguments presents a comprehensive and nuanced depiction of the equitable and just relationship that exists between men and women as prescribed and articulated within the teachings of Islam (Qadri and Siregar, 2023). Consequently, the notion that women are to be viewed as subordinate to men is a socially constructed belief that has emerged over time, which is fundamentally inconsistent with, and indeed stands in stark opposition to, the principles of justice and equality that are so strongly advocated for in Islamic doctrine.

In light of the aforementioned considerations, it is both pertinent and intellectually stimulating to pose a question that seeks to uncover the underlying motivations that contribute to the systematic infringement of the fundamental rights that are inherently entitled to Muslim women. Patriarchal inclinations are frequently referenced by researchers as a significant factor that exacerbates injustices pertaining to the needs and interests of women. Historically, systems characterized by male dominance tend to result in the marginalization of women’s perspectives and the oversight of their requirements across diverse domains of existence, encompassing politics, economics, health, and education. Moreover, the development of Islamic jurisprudence has been significantly influenced by masculine and patriarchal paradigms. Consequently, numerous religious interpretations are distorted by gender bias (Mun’im et al., 2024). In order to effectively confront the pervasive issue of patriarchal dominance within the framework of Islamic societies, a growing number of dedicated Muslim female activists are fervently advocating for immediate and decisive actions that must be taken to promote equality and justice for women. Their endeavor is directed towards the reinterpretation of religious texts and traditions with the objective of eliminating discrimination and ensuring the provision of equal rights. The emphasis is placed on a rigorous analysis of the Quran and Hadith, with the intention of identifying contextual and historical interpretations that sustain gender inequality. These advocates also diligently strive to broaden economic and educational opportunities for Muslim women, accentuating their significance in public spheres. Their initiatives are geared towards the establishment of a society wherein women may achieve their full potential without encountering restrictions predicated on gender (Ismail and Hasan, 2021). Concurrently, the significance of women’s contributions to theology and jurisprudence is being championed, thereby establishing a platform for the development of equitable and inclusive religious standards. The perspectives of women can introduce new dimensions to traditional scriptures, illuminate aspects that have been previously neglected, and even contest entrenched interpretations that have historically been formulated solely by men. In the realm of theology, this may result in a more empathetic and compassionate conception of the divine, prioritizing care and healing over mere authority and retribution (Agustina and Ismah, 2024). The promotion of more expansive discourse and equitable religious practices can bring social transformation that acknowledges and elevates the roles and authority of women. This contribution is essential for the progression of gender equality within Islamic contexts and for augmenting women’s active involvement in religious undertakings (Erwani and Siregar, 2024).

### Religious preachers are raising awareness of women’s rights in social media

Social media is extensively utilized by religious leaders and activists for the dissemination of Islamic teachings. Within their sermons, they engage in discussions concerning a multitude of significant issues pertinent to the lives of Muslims. In light of the ongoing discourse surrounding women’s rights, there has been an initiative to attract the attention of scholars and the general public to this matter via social media platforms. The advent of social media has enabled religious leaders to broaden their audience and connect with individuals who do not frequent mosques or other religious assemblies. This has established a forum to deliberate on various dimensions of Islamic faith and practice, as well as to confront the challenges that Muslims encounter in the contemporary world. The discourse on women’s rights, in particular, has sparked considerable interest and facilitated a more profound comprehension of the diverse perspectives within Islam regarding this issue. Muslim women are progressively utilizing social media platforms to portray their identities, thereby contesting the prevailing stereotypes of oppression and championing their rights (Alfiyah and Ahlan, 2022). The rise of digital platforms has enabled Muslim women preachers to advocate for their rights within the context of Islamic teachings. This trend has achieved significant influence, and they are progressively disseminating our regulations, experiences, and interpretations of sacred texts, encompassing alternative perspectives on matters, rights, and the influence of women in Islam. Through networks, blogs, and online lectures, Muslim women preachers are formulating innovative discourses, striving to uphold the principles of justice, equity, and democracy that are intrinsic to the Islamic tradition. They are actively engaging in the discourse surrounding such pivotal issues as education, employment, familial rights, and political involvement, vigorously defending their decisions and well-founded positions based on a profound understanding of Islamic transformations (Rayhana and Fristyarini, 2024).

For example, South East Asian religious authorities present divergent perspectives on gender equality, illustrating the plurality of thought within the Islamic discourse. Their sermons focused on upholding the guidelines rooted in Islamic principles and the values pertaining to women. Through their speeches, they strive to serve as agents of transformation by grounding their arguments in the Islamic viewpoint (Parwanto et al., 2024). The female bloggers hailing from Western countries are increasingly articulating their perspectives regarding the rights of women through their various online preaching endeavors, thereby contributing significantly to the discourse surrounding this vital subject. In their discourse, they emphasize the profound role that Islam plays in enhancing the status of women within society and advocate for a thorough reconsideration of contentious topics such as polygamy; furthermore, they actively challenge their male counterparts who seek to undermine the rights that have been divinely ordained by Allah for women. The sermons and discussions that they disseminate through the internet have garnered a notably positive reception from their female audience, as they effectively raise awareness on this complex and often debated issue, encouraging further dialogue and reflection. Overall, these activities serve not only to empower women but also to foster a deeper understanding of their rights and the teachings of Islam, which can lead to a more equitable society for all (Kurmanaliyev et al., 2024). The prominent Kazakh preacher Nurlan Baizhigitov serves as a strong example in this regard. Against the backdrop of nationwide cases of women’s abuse and oppression, he became a vocal advocate for the protection of women’s rights. He dedicated his sermons and live broadcasts to this critical issue in order to draw public attention. In his online preaching, he emphasized the problem of domestic violence and sharply criticized male abusers. His preaching activities gained significant popularity, and he was soon labeled a “progressive Muslim preacher” in Kazakhstan (Kerim et al., 2025).

The matter of women’s rights is currently the subject of fervent discourse among male clergy during their online sermons. They implore male audiences to honor the rights of women, to accord them the respect they deserve, and, most crucially, to prioritize Islamic tenets over their patriarchal perspectives and customs. These sermons frequently articulate compelling arguments derived from the Quran and Hadith, underscoring the significance of treating women with fairness and respect in alignment with Islamic doctrines. Sermons of this nature assume a pivotal role in transforming public consciousness and dismantling deeply embedded patriarchal paradigms. They emphasize that the advocacy for women’s rights transcends being solely a women’s concern, but rather constitutes an essential component of Islamic ethics and morals that pertains to all individuals within society. Consequently, within the Muslim community, there is a profound shift in the dialogue concerning the protection of women’s rights as prescribed by Islamic law (Kerim and Nusipbayev, 2024). The engagement of online religious leaders significantly broadens the avenues for the propagation of religious doctrines pertaining to the rights of women. Ultimately, the proactive participation of online religious leaders in the discourse surrounding women’s rights serves to elevate awareness and transform societal perspectives. This, in turn, has the potential to facilitate an enhancement in the status of Muslim women across various domains of existence, ranging from education to involvement in public, economic and political arenas.

### Actions aimed at restoring women’s rights

The protection of the rights of Muslim women represents a complex undertaking that necessitates an integrated approach encompassing various domains: legislation, education, socio-economic development, and cultural awareness. On the international stage, initiatives are being undertaken to ratify and implement conventions aimed at safeguarding women’s rights. In national contexts, numerous countries are engaged in the formulation and execution of laws designed to combat violence against women while ensuring equitable access to education, healthcare, and employment opportunities. Special emphasis is placed on issues pertaining to marriage and family, including the prevention of forced marriages and the safeguarding of women’s rights in divorce proceedings. Educational programs serve a pivotal role in enhancing awareness regarding women’s rights and in challenging prevailing stereotypes (Meidina, 2022). The support of women’s organizations and initiatives constitutes a vital aspect of the endeavor to protect the rights of Muslim women. This encompasses the support of progressive religious scholars and activists who champion women’s rights within the framework of the Islamic tradition. The struggle for the rights of Muslim women must take into consideration the specific contexts and challenges they encounter in various regions of the world (Ferdousi, 2021).

The safeguarding of women’s rights against domestic violence constitutes a significant concern within the framework of Islamic jurisprudence. Islam, as a holistic system, profoundly underscores the principles of justice and compassion, highlighting the necessity to safeguard all constituents of society, particularly women. The Quran and Sunnah provide unequivocal directives against any form of violence and maltreatment, advocating for the respectful and dignified treatment of wives. Numerous Islamic scholars contend that domestic violence fundamentally contradicts the core tenets of Islam. They interpret religious texts in a manner that upholds women’s entitlement to a secure and dignified existence. In instances where violence transpires, victims are entitled to protection, legal recourse, and restitution (Az-Zahra et al., 2024). Islam categorically forbids any manifestation of violence directed towards women. The Islamic scriptures, including the Quran and Sunnah, provide unequivocal guidance on the necessity of treating women with utmost respect and compassion. Any form of violence, whether it be physical, psychological, or economic in nature, is antithetical to both the essence and the stipulations of Islamic jurisprudence.

The prohibition of forced marriages by Muslim clerics signifies a significant advancement in the initiative to safeguard women’s rights. This measure seeks to abolish a practice that infringes upon fundamental human rights, notably the right to autonomously select a spouse. Forced marriages frequently result in grave repercussions for women, depriving them of the opportunities to pursue education, engage in employment, and fully participate in public life. Religious authorities disseminate fatwas that categorically forbid parents from arranging marriages for their daughters without their consent. These fatwas, grounded in Islamic texts and principles, call to respect a woman’s agreement in the institution of marriage. Forced marriage is regarded as a profound violation of human rights and is inconsistent with the essence of Islam, which advocates for mutual respect and harmony between spouses (Halim et al., 2024). Most Islamic scholars elucidate that a marriage executed without the bride’s consent is rendered invalid. Parents are not entitled to impose a marriage partner upon their daughters, even if they perceive it to be in their best interests. It is asserted that the decision to enter into marriage must be predicated upon the free and informed choice of a woman. The esteemed scholars are urging Muslim communities to adhere to these fatwas and ensure that women’s rights in matrimonial matters are duly honored. The various educational programs and information campaigns are designed to enhance awareness regarding the unacceptability of forced marriages (Agustina and Ismah, 2024).

To duly safeguard the rights of women within the context of polygamy as prescribed by Islamic law, it is imperative to mention the significance of equitable treatment among co-wives. The Quran’s resounding call for justice and equity mandates that husbands treat all wives with fairness and impartiality. Educating women about their rights under Islamic law becomes a potent instrument, amplifying their voices within the marriage. This empowerment enables them to negotiate agreements that ensure shared support and mutual responsibility among co-wives. Such a strategy not only fosters a more harmonious and balanced family dynamic but also challenges entrenched patriarchal interpretations that often marginalize women’s roles (Motiejūnė, 2025).

In the exploration of the educational rights and status of Muslim women, it is imperative to acknowledge that their access to education not only serves to empower them on an individual level but also acts as a catalyst for comprehensive societal transformation. Historically, women who have attained education have assumed crucial roles across diverse domains, making substantial contributions to cultural, scientific, and political progress within their respective communities (Saepudin and Zahra, 2023). The females such as Fatima al-Fihri, who established one of the most ancient universities globally, illustrate how the education of women has historically nurtured intellectual advancement and social reform. Nonetheless, in spite of these profound legacies, contemporary obstacles remain as numerous Muslim-majority nations continue to confront gender inequities in education, which are frequently intensified by deep-rooted patriarchal conventions. Addressing this disparity necessitates a unified endeavor to harmonize educational policies with Islamic principles that promote equal rights and opportunities for both sexes, thereby ensuring that future generations of women are able to fully achieve their potential and make meaningful contributions to society (Jawed and Sikka, 2024). Education is an essential necessity for Muslim women, as they serve as the conduit for imparting Islamic knowledge to a female audience. This responsibility encompasses not only a profound comprehension of religious texts but also the proficiency to adapt these texts for the education and development of girls and young women. Muslim female scholars and activists hold a pivotal position in shaping the religious perspective and moral values among the women. In the pursuit of advancing women’s rights, several Muslim nations are establishing councils comprised of women scholars (Junaidi et al., 2022).

Sharia courts are presently engaged in the protecting of women’s rights through the issuance of fatwas. In various Muslim nations they are reassessing their rulings to guarantee that wives are adequately protected post-divorce. This step has significantly enhanced the circumstances of second wives, who were formerly left destitute after divorce (Analiansyah et al., 2024). Besides, the sharia courts increasingly adjudicating cases pertaining to domestic violence and coerced marriages, rendering judgments that favor the protection of women. Rulings promulgated by these courts advocate for the respect of women’s rights and denounce any form of discrimination or violence against them. Ultimately, sharia courts fulfill a vital function in the advancement and protection of women’s rights within Muslim communities, ensuring that Sharia principles are maintained in light of contemporary challenges and requirements (Ferdousi, 2021).

Some Islamic organizations call for heightened focus on disability matters within contemporary society and highlight their active involvement in societal affairs and the prevention of social disparities. In Indonesia, in order to tackle the contemporary challenges pertaining to disability rights, two prominent Islamic organizations, Nahdlatul Ulama (NU) and Muhammadiyah, have drafted documents to support disabled women. They signify a pivotal advancement in the evolution of Islamic human rights movement, aimed at addressing modern exigencies and fulfilling the requirements of individuals. The objectives delineated in these documents furnish additional guidance and principles, promoting the dignity of individuals with disabilities, while safeguarding their rights and opportunities. The issuance of such fatwas serves as a testament to the increasing cognizance of the significance of inclusion and social equity within the Muslim community (Maftuhin, 2023).

As part of the realization of women’s rights, several Muslim nations are establishing councils comprised of women scholars. This represents a significant advancement towards the development of a robust Islamic society, wherein the voices of women are not merely acknowledged but also actively engaged in the formulation of religious and social norms. The creation of such councils evidences the acknowledgment of the vital role that women’s intellectual contributions play in the evolution of Islamic thought. It paves new avenues for women scholars, enabling them to disseminate their knowledge and experiences, exert influence over the creation of policies and legislation within their respective countries. This also fosters a more balanced and inclusive approach to addressing matters pertaining to the rights and responsibilities of Muslims (Rohmaniyah et al., 2022). With the foundation of religious tenets, female ulamas possess the capacity to engage in meaningful discourse regarding women’s issues and to mitigate various instances of gender injustice. The involvement of female ulamas in the expansion of social issues enhances the dimensions of religious dialogue, introducing a feminine perspective and strengthening a more profound comprehension of the challenges confronting women in society. They can serve as intermediaries between religious doctrines and contemporary realities, facilitating the discovery of solutions that align with both religious values and the exigencies of modern society (Mulia, 2013).

Male religious authorities also play a significant role in the discourse surrounding women’s rights. These contributions are frequently complex, integrating traditional interpretations of sacred texts with modern perspectives on gender equality. Certain religious leaders actively champion the cause of women’s rights within their respective faiths, highlighting the principles of justice, compassion, and respect for human dignity that they assert form the foundation of religious teachings. They may initiate discussions aimed at challenging antiquated norms and practices that negatively affect women’s rights, thereby fostering more inclusive interpretations of sacred texts. Conversely, other religious leaders adopt a more conservative approach, advocating for the maintenance of traditional gender roles while simultaneously acknowledging the necessity of safeguarding women from violence and discrimination. Irrespective of their particular stance, the involvement of religious leaders in the discourse on women’s rights can significantly influence public opinion and facilitate positive transformation (Al-Sharmani, 2023). Tahar al-Haddad serves as an exemplary model of such religious figures. He emerged as a prominent intellectual, author, reformer, and social activist within North Africa, particularly in Tunisia. He is historically esteemed as a courageous advocate for women’s rights in the Muslim context and a supporter of social progress. His preaching activism substantially impacted the development of the feminist movement and the modernization of legal systems in Tunisia and beyond. He championed a reevaluation of traditional norms and customs, which he perceived as obstructing the nation’s advancement and restricting women’s rights (Ismail and Hasan, 2021).

The issue of women’s rights in Muslim countries requires a comprehensive and careful approach that takes into account cultural, social, and societal factors. A viable solution to this problem can only be realized if both Muslim women and men collaborate effectively. Muslim women play a crucial role in this context, as they have direct experience and understanding of the existing challenges. Their viewpoints must be recognized and incorporated into the development of strategies and initiatives aimed at improving their position in society. Nevertheless, to achieve lasting change, the participation and support of Muslim men are crucial. They must reevaluate traditional roles, promote women’s education and economic empowerment, and actively fight against oppression and violence toward women. Cooperative efforts between men and women, based on the ideals of justice, freedom, and democracy for women within Islam, are essential for creating an equitable and prosperous society where the rights and liberties of every woman are protected and promoted.

## Conclusion

The study unveiled that Muslim women during the Islamic medieval period held considerable rights and status, which stands in stark contrast to the difficulties that numerous Muslim women encounter today. This era witnessed a flourishing of female voices and contributions, reflecting a more inclusive interpretation of Islamic principles. However, over time, socio-political factors and cultural interpretations led to a gradual erosion of these rights and freedoms. The rise of patriarchal norms, coupled with misinterpretations of religious texts, resulted in discriminatory practices that marginalized women in many Muslim societies. The article emphasizes that even though Islam bestows certain rights, numerous women continue to encounter discrimination and socio-cultural hurdles that impede their ability to access education, employment, and political engagement. The ongoing influence of traditional and patriarchal norms presents considerable challenges that must be confronted to attain genuine gender equality. The current disparities highlight the need to revisit and reclaim the original spirit of Islamic teachings regarding gender equality. By promoting education, critical thinking, and a deeper understanding of religious texts, it is possible to challenge harmful interpretations and empower women to assert their rights and realize their full potential. Contemporary Muslim scholars and activists are working to revive the progressive aspects of Islamic tradition, advocating for equal rights, access to education, and participation in all spheres of life. Their efforts aim to create a more just and equitable society that reflects the true essence of Islamic values.

The article further details the initiatives undertaken to enhance the safeguarding of women’s rights within the framework of Islamic law. These initiatives encompass engaging religious leaders to actively address the topic, enacting laws and fatwas that elevate the status of women. The involvement of prominent leaders is crucial in elevating the visibility and commitment to women’s rights. Influential preachers can articulate the provisions of Islamic law, promote awareness of women’s rights, and stress the importance of adhering to them. These regulations can impact various facets of women’s lives, including their rights to education, employment, training, and divorce. The amalgamation of these measures fosters a conducive environment for the safeguarding and advancement of women’s rights in Muslim communities. The research calls for a collective effort to dismantle the structural barriers that impede women’s advancement. It emphasizes the importance of societal support and understanding of women’s rights to facilitate change and promote justice within Islamic societies.

The discourse surrounding women’s rights within the Islamic context is multifaceted and necessitates a comprehensive approach that encompasses sustained efforts, a long-term commitment, and decisive actions. Understanding the intricate interplay of cultural, religious, and social factors is essential for fostering an environment that promotes gender equality and empowers women. As we navigate this complex landscape, it is imperative to engage in open dialogue, challenge prevailing stereotypes, and advocate for reforms that align with the core principles of justice and equity inherent in Islamic teachings. Ultimately, achieving progress in women’s rights within this framework will require collaboration among scholars, activists, and community leaders to ensure that women’s voices are heard and their rights upheld.

## Data Availability

The original contributions presented in the study are included in the article/supplementary material, further inquiries can be directed to the corresponding author.

## References

[ref1] Abdullah F. Hamzah G. (2024). Al-Qur’ān and the humanization of women (analysis of women’s rights and position in the verses of the Qur’an). Tawasut Indonesian J. Moderate Islam 11:12303. doi: 10.31942/ta.v11i2.12303

[ref2] Agustina A. Ismah N. (2024). Challenging traditional Islamic authority: Indonesian female Ulama and the fatwa against forced marriages. J. Islamic Law 5, 125–146. doi: 10.24260/jil.v5i1.2319

[ref3] Ahmed S. (2022). Women in Islam their rights and jurisprudential attachments –an objective study according to the glorious Qur an and Sunnah. J. Sci. Dev. Stud. Res. 3, 87–100. doi: 10.61212/jsd/52, 41231662

[ref4] Alfiyah A. Ahlan A. (2022). Eksistensi Perempuan Dalam Dakwah Kontemporer Perspektif Al-Qur’an Dan Hadis. Madinah: Jurnal Studi Islam 9:1386. doi: 10.58518/madinah.v9i2.1386

[ref5] Al-Sharmani M. (2023). Islamic feminist hermeneutics: between scholarship and lived realities. J. Fem. Stud. Relig. 39, 95–97. doi: 10.2979/jfemistudreli.39.2.15

[ref6] Analiansyah A. Dhiaurrahmah D. Jamhuri J. Salam A. J. Iskandar M. (2024). Polygamy and women’s rights: an examination of divorce litigation in sharia court rulings pertaining to revisions in Indonesian matrimonial legislation. Al-Istinbath Jurnal Hukum Islam 9, 761–786. doi: 10.29240/jhi.v9i2.10833

[ref7] Arfah A. (2024). The position of women in perspective of Islamic history: dismissed the issue of inequality in Islam. Indones. J. Islamic Hist. Cult. 5, 21–27. doi: 10.22373/ijihc.v5i1.4798

[ref8] Az-Zahra A.’i. Zuhdi S. Rahmawati U. D. Dewi T. P. (2024). Protection of women’s rights against cases of domestic violence from an Islamic perspective. Jurnal Al-Hakim: Jurnal Ilmiah Mahasiswa, Studi Syariah, Hukum Dan Filantropi 6, 231–242. doi: 10.22515/jurnalalhakim.v6i2.10163

[ref9] Begum M. S. I. Ismail I. Yaakob Z.’a. Razick A. S. Abdullah M. M. A. (2024). Gender equity in Muslim family law: modern and contemporary ‘Ulamā’s view. Al-Ahkam 34, 221–256. doi: 10.21580/ahkam.2024.34.2.20773

[ref10] Belhachmi Z. (2005). Al-salafiyya, feminism and reforms in twentieth century Arab-Islamic society. J. North Afr. Stud. 10, 111–141. doi: 10.1080/13629380500252119

[ref11] Bishin B. G. Cherif F. M. (2017). Women, property rights, and islam. Comp. Polit. 49, 501–519. doi: 10.5129/001041517821273026

[ref12] Erwani I. Siregar A. S. (2024). The role of women in Islamic sacred texts: a critical study of women’s narratives and authority in Islamic tradition. Pharos J. Theol. 106.1:1–14. doi: 10.46222/pharosjot.106.6

[ref13] Ferdousi N. (2021). Protection of wife’s right to maintenance in Bangladesh: an overview. Malays. J. Shariah Law 9, 173–180. doi: 10.33102/mjsl.vol9no2.246

[ref14] Firdaus D. Arifin Z. (2018). Pendidikan Perempuan Perspektif Quraish Shihab Dalam Tafsir al Misbah. Tribakti Jurnal Pemikiran Keislaman 29, 208–234. doi: 10.33367/tribakti.v29i2.595

[ref15] Gutmann J. Voigt S. (2015). The rule of law and constitutionalism in Muslim countries. Public Choice 162, 351–380. doi: 10.1007/s11127-015-0237-z, 41439200

[ref16] Hafizah Y. (2024). Gender and Islam through Leila Ahmed’s lens: methodology, social realities, and personal insights. Muadalah 12, 129–141. doi: 10.18592/muadalah.v12i2.12467

[ref17] Halim A. R. Al Amruzi M. F. Jalaluddin J. (2024). Legal formulation for forced marriage prevention through the decision of Wali Mujbir in religious courts and its relevance with Maqȧṣid Syari‘ah and human rights. Mazahib 23, 79–116. doi: 10.21093/mj.v23i1.6189

[ref18] Hessa A. (2021). Women and community services in Islamic history. J. Al-Tamaddun 16, 49–57. doi: 10.22452/JAT.vol16no2.4

[ref19] Ismail Z. Hasan M. R. (2021). Islamic legal modernism and women’s emancipation in Tunisia. Mazahib 19, 281–314. doi: 10.21093/mj.v19i2.2800

[ref20] Jawed A. Sikka G. (2024). Educational rights and status of Muslim women as provided in Islam. Int. J. Multidiscip. Res. 6:25315. doi: 10.36948/ijfmr.2024.v06i04.25315

[ref21] Junaidi M. Mahmutarom M. Ekaningrum I. R. Suryani K. (2022). Education for women in the global era of Islamic perspectives. Qalamuna Jurnal Pendidikan Sosial Dan Agama 14:4790. doi: 10.37680/qalamuna.v14i1.4790

[ref22] Kerim S. Kurmanaliyev M. Ongar Y. Kaliyeva Y. (2025). Digital transformation of Islamic preaching in Kazakhstan: identifying famous online preachers and their influence. Millah J. Relig. Stud. 24, 611–644. doi: 10.20885/millah.vol24.iss2.art2

[ref23] Kerim S. Nusipbayev E. (2024). The use of social networks in Islamic preaching. Bullet. KazNU Ser. Relig. Stud. 37, 3–12. doi: 10.20885/millah.vol24.iss2.art2

[ref24] Khan A. (2021). “The role of Islam in establishing women’s rights in the Muslim world.” honors undergraduate theses 950. Orlando: University of Central Florida. Available online at: https://stars.library.ucf.edu/honorstheses/950

[ref25] Khanom J. Islam M. T. Mahmudulhassan (2024). Women’s rights in Islamic culture: a bibliometric analysis of trends, influential authors, and institutional contributions (1969–2023). Solo Univ. J. Islamic Educ. Multicult. 3, 27–42. doi: 10.61455/sujiem.v3i01.232

[ref26] Khofiyya R. N.'a. Tazkiyatan N. Darnoto D. (2024). The roles and rights of women in the modern era from an Islamic perspective. Hayula Indones. J. Multidiscip. Islamic Stud. 8, 215–226. doi: 10.21009/hayula.008.02.05

[ref27] Kurmanaliyev M. Kerim S. Almukhametov A. Amankul T. (2024). American and European Muslim female bloggers increase their preaching efforts in social media. Religion 15:1485. doi: 10.3390/rel15121485

[ref28] Lo S. Seltzer R. (2015). Gender in the midst of change: examining the rights of Muslim women in predominately Muslim countries. J. Int. Womens Stud. 16:4. Available online at: https://vc.bridgew.edu/jiws/vol16/iss2/4

[ref29] Maftuhin A. (2023). Islamic law, disability, and women in Indonesia: the cases of Nahdlatul Ulama and Muhammadiyah. J. Disabil. Relig. 28, 13–27. doi: 10.1080/23312521.2023.2255860

[ref30] Maloko M. T. Chotban S. (2024). Perlindungan Hak Asasi Perempuan Dalam Hukum Islam protection of women’s rights in Islamic law. Al-Ahkam 6:2632. doi: 10.47435/al-ahkam.v6i1.2632

[ref31] Meidina A. R. (2022). Meninjau Ulang Iwadl Khuluk Perspektif Keadilan Gender. Al-Manahij: Jurnal Kajian Hukum Islam 16, 77–90. doi: 10.24090/mnh.v16i1.6027

[ref32] Motiejūnė G. S. (2025). Polygamy in Islam: a study on its religious justifications and empowerment of women within Islamic teachings. QiST J. Quran Tafseer Stud. 4, 59–74. doi: 10.23917/qist.v4i1.6948

[ref33] Mulia M. (2013). Hukum Islam Dan Dinamika Feminisme Dalam Organisasi Nahdlatul Ulama. Al-Ahkam 23, 37–56. doi: 10.21580/ahkam.2013.23.1.72

[ref34] Mun’im Z. Nasrudin M. Suaidi S. Hasanudin H. (2024). Revisioning official Islam in Indonesia: the role of women ulama congress in reproducing female authority in Islamic law. AHKAM J. Ilmu Syariah 24:34744. doi: 10.15408/ajis.v24i1.34744

[ref35] Panjwani I. (2020). The ignored heritage of Western law: the historical and contemporary role of Islamic law in shaping law schools. Law Teach. 54, 562–577. doi: 10.1080/03069400.2020.1827841

[ref36] Parwanto W. Sulaiman S. Riyani R. (2024). Gender in social media: a comparative study of Abd. Somad and Adi Hidayat’s interpretation on gender equality. Jurnal Ilmiah Widya Borneo 7, 91–102. doi: 10.56266/widyaborneo.v7i2.330

[ref37] Qadri B. Siregar I. M. (2023). Islamic renewal in the field of family law: a historical analysis of equality. El-Usrah: Jurnal Hukum Keluarga 6, 444–455. doi: 10.22373/ujhk.v6i2.17128

[ref38] Rayhana S. N. Fristyarini A. (2024). Revolution in religious propaganda: unlocking the potential of digital dawah and its limits for Muslim women activists. Cendekia 10, 195–213. doi: 10.37348/cendekia.v10i2.354

[ref39] Rizal D. Irman I. Putri D. Miftahurrahmah M. Yustiloviani Y. Kamaluddin K. (2024). Reinterpreting religious texts on gender equality: the perspective of Ahmad Syafii Maarif. JURIS (Jurnal Ilmiah Syariah) 23, 327–336. doi: 10.31958/juris.v23i2.10233

[ref40] Rohmaniyah I. Kotele S. Pabbajah M. Rasyidah H. S. (2022). Female ulama’s authority: deconstructing masculine domination in Islamic norms and practices. Int. J. Islamic Thought 6, 54–61. doi: 10.24035/ijit.21.2022.225

[ref41] Saepudin D. Zahra N. H. (2023). Women in Islamic historiography and history. J. Indo-Islamika 12, 87–101. doi: 10.15408/jii.v12i2.28976

[ref42] Samadi M. (2021). Advancing the legal status of women in Islamic law. Leiden, The Netherlands: Brill | Nijhoff.

[ref43] Warren D. (2011). Women in classical Islamic law: a survey of the sources. Br. J. Middle East. Stud. 38, 449–451. doi: 10.1080/13530194.2011.621717

